# Videoconferencing for Home Care Delivery in Japan: Observational Study

**DOI:** 10.2196/23539

**Published:** 2021-09-01

**Authors:** Hirotomo Miyatake, Makoto Kosaka, Satoshi Arita, Chie Tsunetoshi, Hidehisa Masunaga, Yasuhiro Kotera, Yoshitaka Nishikawa, Akihiko Ozaki, Hiroyuki Beniya

**Affiliations:** 1 Orange Home-Care Clinic Fukui Japan; 2 Faculty of Medicine The University of Tokyo Tokyo Japan; 3 College of Health, Psychology and Social Care University of Derby Derby United Kingdom; 4 Department of Breast Surgery Jyoban Hospital of Tokiwa Foundation Iwaki Japan; 5 Medical Governance Research Institute Tokyo Japan

**Keywords:** telehome care, videoconference, home care, caregiver, telepresenter, mobile phone

## Abstract

**Background:**

Telemedicine has been increasingly used in many health care fields, including home care, where patients receive medical care at home. Owing to the current COVID-19 crisis, the value of telemedicine via videoconferencing is more recognized, particularly in allowing immobile patients to continue receiving care. However, the efficacy of telemedicine in home care settings in Japan remains to be fully appraised.

**Objective:**

This study aims to identify the use and impact of telemedicine in a singular home care delivery setting in Japan.

**Methods:**

A retrospective observational study was conducted using patient and other administrative records from a home care clinic. We considered patients who were involved in videoconferencing with home care physicians and telepresenters serving patients during 2018 and 2019. We extracted sociodemographic data of the patients and details of the videoconferencing and descriptively illustrated some specific cases.

**Results:**

In a home care clinic in Japan, videoconferencing was conducted in 17 cases (involving 14 patients) over a 2-year period. Of all the cases, 12% (2/17) required emergency transfers and were hospitalized. A total of 88% (15/17) of cases remained; 71% (12/17) of cases were found to need extra medication or to go to a medical facility for consultation, whereas 18% (3/17) of cases were found not to be in need of urgent attention and were asked to rest. Problematic symptoms subsequently improved in 82% (14/17) of cases, and only 6% (1/17) of cases were later hospitalized.

**Conclusions:**

Telemedicine was deemed effective for assessing patients’ conditions in the home care setting in situations where home visits by a physician cannot be carried out. Our findings indicate that consultations via videoconferencing are safe and effective, suggesting more active use of videoconferencing in other clinical contexts.

## Introduction

### Background

Telemedicine, the use of information and communication technology to deliver health care at a distance, has been increasingly used to deliver health care covering a wide range of specialties, for numerous conditions, and through a variety of channels and systems [1]. An ever-increasing body of evidence suggests that care delivered via telemedicine is safe and can be as, if not more, effective and economical as face-to-face care [1]. However, one review article pointed out that the effectiveness of telemedicine could depend on conditions and specialties, and evidence on its cost-effectiveness was limited [2]. In addition, it has been suggested that applications of telemedicine in home care should be cautiously conducted, holistically weighing various individual characteristics and external factors surrounding patients [3].

Moreover, significant barriers to wider use have been identified: health care providers and patients face regulatory ambiguities and financing uncertainties. Inequalities in both health and digital literacy are another concern, as patients who could benefit the most are often those who are the least able to have access to or make use of telemedicine [4]. Telemedicine has evolved to help improve effectiveness, efficiency, and equity in health care. However, it can also create risks, amplify existing inequalities, and worsen health care delivery.

Japan has been actively promoting telemedicine as a means to help meet the evolving health care needs of the population. Key areas of focus have been (1) access to health care for rural and remote communities, (2) home telemedicine, and (3) illness prevention and lifestyle modification. Evidence on the safety and efficacy of telemedicine in various situations has been presented at conferences [5]. The Japanese government has been encouraging telemedicine, especially as part of a strategy focusing on an effective home-based primary care approach, in an attempt to move away from the nation’s famed lengthy hospitalization practice [6]. In 2015, Japan’s Ministry of Health, Labor and Welfare (MHLW) permitted the use of telemedicine in areas other than rural and remote areas [7] and, in 2018, allowed medical treatment fees for remote examinations to be partially covered under the national health insurance scheme. However, provider-to-patient telemedicine services are only permitted after an initial face-to-face meeting has taken place between the physician and the patient [8]. It is the sole responsibility of the physician to decide whether remote health care is safe and appropriate.

### Objectives

Only a few telemedicine studies in Japan have been published, mostly in the Japanese language, limiting the global reach of the findings [9]. An in-depth appraisal of the efficacy of telemedicine is particularly important in home care settings, as there are many factors that could prohibit physicians from physically visiting patients’ homes immediately, beyond planned visits and in emergency situations. Telemedicine via videoconferencing is a possible means to help overcome this problem. However, its effectiveness and safety in assessing patients’ conditions, including identifying potential acute illnesses and potential health emergencies, has not been evaluated in the home care setting in depth. Accordingly, the main goal of this study is to demonstrate some cases using videoconferencing and discuss its efficacy and safety and to identify some key elements in the home care setting in Japan.

## Methods

### Overview of Japanese Health Insurance System, Home Care, and Remote Examination

Home care in Japan is provided and paid for within the Japanese health insurance system. The Japanese government has promoted home care as a part of the Integrated Community Care System, which requires cooperation between home care providers [10] to reduce medical costs and overcome constraints, such as the number of physicians in Japan (2.5 per 1000 inhabitants), which is the lowest among the Organization for Economic Cooperation and Development countries [11].

### Integrated Community Care System

The Integrated Community Care System, which is designed to provide medicine, nursing, and life support in an integrated format, has been developed to enable some patients, particularly older adults, to continue living at home in the manner to which they have become accustomed [10]. Home care is indicated only when the physician determines that it is applicable. There are no clear criteria, but *patients who are at least unable to walk to the clinic on their own without the help of family members or caregivers* is a requirement, and there are no age restrictions. Therefore, the patients involved in this research were those living in specialized housing facilities for older adults where a formal caregiver was available and those who were regularly visited by their physician, living in their own residences with the assistance of informal family caregivers.

### Cooperation Between Home Care Physicians and Nurses and Pharmacists

In Japanese home care, patients receive nursing services from institutional nurses or visiting nurses under the medical insurance system or the long-term care insurance system. Some specialized caregiving facilities use full-time institutional nurses. Visiting nurses belong to clinics, hospitals, or nurse stations and provide physical and mental health services, including medical care and basic life support services. However, any physical intervention, such as management of intravenous injections or blood sampling, can only be administered under the supervision of the patient’s physician. The clinic provides a visiting nurse service; however, it is up to patients whether they make use of the service.

In addition, pharmacies are counted as facilities that provide medical services in the Integrated Community Care System. Some pharmacies or clinics dispatch community pharmacists and provide indications for drug management to home care patients [11].

### Remote Examination in the Japanese Home Care Setting

The MHLW has created strict definitions for what constitutes a *remote examination*, in which physicians deal with patients via a digital audiovisual link, such as via a videophone or the internet. Remote examinations were conducted in the same manner as face-to-face examinations. Until recently, the first examination must have been face-to-face; however, because of COVID-19, remote examination can now be used as the first examination session under certain conditions. As of April 2020, all physicians who intend to use videoconferencing are required to undertake training regulated by the MHLW.

Doctor to patient with nurse has been introduced as a common form of implementing remote examinations, in which a physician can see patients receiving home care via a digital audiovisual connection but only with a visiting nurse attending the patient. In this situation, visiting nurses are supposed to provide medical assistance within the range expected in the treatment plan and visiting nursing instruction. In this study, all remote examinations were conducted as a substitute for unplanned physician visits. Although doctor to patient without nurse is also permitted as a form of remote examination, most of the informal caregivers or patients involved in this study requested the presence of nurses.

In Japan, physicians are primarily responsible for any intervention, either directly or in an oversight capacity, conducted as part of any remote examination or intervention. If a physician determines that they are unable to perform an adequate remote examination, the physician must immediately discontinue the remote examination and arrange a face-to-face examination. Physicians are also required to take adequate security measures to ensure that patients’ medical information is not compromised.

### Setting and Participants

The Orange Home Care Clinic specializes in providing medical care to patients based on their own homes or residences in Fukui City, Japan. This clinic has five working teams, consisting of 1 physician and 1 clerk per team, treating an average of 330 patients annually. Several nurses have been employed to support the smooth operation of the clinic, such as coordination with other visiting nurse stations. Therefore, regular nursing care is provided by other visiting nurse stations. Each team visits 8-10 patients a day on weekdays. The visiting area covers a radius of 16 km from the clinic. Owing to various unforeseen circumstances (eg, natural disasters), the clinic used videoconferencing in 17 instances from January 2018 to December 2019.

### Data Collection

Basic information on patients, such as age, sex, primary diseases, and availability of caregivers, was collected from medical records at the clinic. Data on cases where videoconferencing was used, such as the reason for the consultation, the telepresenter, any intervention, and the outcome, were collected from medical records and other documentation.

The availability of a caregiver was categorized as (1) formal caregiver (a patient living in a specialized housing facility where an official caregiver is present), (2) informal caregiver (patient living with a family member), and (3) no caregiver (a patient living alone). We used this classification because the feasibility of videoconferencing is supposed to depend on the living environment and the demographics in Japan have been changing rapidly, and the number of single-person households, especially among older adults, has been increasing significantly [12].

In this study, videoconferencing was performed when a caregiver or a visiting nurse decided that it was necessary to consult with a physician when visiting the patient. Videoconferencing was performed as an alternative to an emergency home visit because the physician could not visit immediately. The reason for videoconferencing consultation involved the chief complaint when a consultation was requested by the patient or caregiver. The telepresenter was the person with the patient who orchestrated the remote videoconferencing connection with the physician. Telepresenters need to have adequate knowledge and skill with respect to information technology devices and a proper understanding of the patient’s status. They can be informal caregivers, formal caregivers, and visiting nurses; however, we defined the telepresenter as *no one* when the patients facilitated videoconferencing by themselves. The intervention referred to any medical procedure, which was determined to be necessary during videoconferencing and before the next planned visit could be undertaken. Although the options of interventions depend on the presence of nurses with patients, interventions such as applying drugs to patients are applicable even if patients contact the physician by themselves. The outcome was categorized as (1) symptom improvement when the patients did not need additional treatment by physicians other than intervention decided through videoconferencing, (2) patients required another unplanned visit by a home care physician before the next planned visit, or (3) patients required hospitalization (when the patient was hospitalized after videoconferencing).

### Ethical Review

This study adheres to the Ethical Guidelines for Medical and Health Research Involving Human Subjects and meets Japan’s local legal requirements. Study approval was granted by the Ethics Committee of the Medical Governance Research Institute on June 4, 2020 (MG2018-18-20200604).

## Results

### Participant Statistics

Table 1 shows a summary of all the cases included in this study.

**Table 1:** Sociodemographic details of the patients and videoconference information.

ID	Age (years)	Sex	Primary disease	Availability of caregiver	Reasons for remote consultation	Telepresenter	Diagnosis	Intervention	Outcome
1-1	97	Female	Stomach cancer	Formal caregiver	Fall	Visiting nurse	Suspect of bone fracture	Prescription of pain reliever	Symptom improvement
1-2	97	Female	Stomach cancer	Formal caregiver	Phlegm	Visiting nurse	Bacterial infection	Prescription of antibiotics	Symptom improvement
2	92	Female	Dementia	Formal caregiver	Vomiting	Visiting nurse	Gastroenteritis	Prescription of antiemetic drug	Symptom improvement
3	93	Male	Dementia	Informal caregiver	Scratch	Visiting nurse	Eczema	Applying ointment	Symptom improvement
4	70	Female	Last stage of stomach cancer	Informal caregiver	Headache	Visiting nurse	Muscle contraction headache	Treatment in acupuncture clinic	Symptom improvement
5	66	Female	Last stage of lung cancer	Informal caregiver	Uncomfortable feeling	Visiting nurse	Dehydration	Infusion	Symptom improvement
6	35	Male	Cerebral contusion	Informal caregiver	Suspect of drug eruption	Visiting nurse	Drug eruption	Stopping antibiotics	Symptom improvement
7	33	Male	Acquired cerebral palsy	Informal caregiver	Suspect of low- temperature burn	Informal caregiver	Low-tempera- ture burn	Applying ointment	Symptom improvement
8	16	Male	Neurofibromato- sis type 1	Informal caregiver	Fever	Visiting nurse	Acute bronchitis	Prescription of antibiotics	Symptom improvement
9	11	Male	West syndrome	Informal caregiver	Epileptic seizures	Institutional nurse	Epileptic seizures	Watch and wait	Symptom improvement
10	7	Male	Cerebral palsy	Informal caregiver	Swelling of the eyelid	Institutional nurse	Hordeolum	Prescription of eye-drops	Symptom improvement
11-1	4	Female	Double outlet right ventricle	Informal caregiver	Suspect of CV^a^ block	Visiting nurse	CV block	Emergency transfer	Hospitaliza- tion
11-2	4	Female	Double outlet right ventricle	Informal caregiver	Vomiting and diarrhea	Visiting nurse	Dehydration	Emergency transfer	Hospitaliza- tion
12	3	Female	Lissencephaly	Informal caregiver	Tachycardia	Visiting nurse	None	Watch and wait	Symptom improvement
13-1	2	Male	Cerebral palsy	Informal caregiver	Increase of phlegm	Informal caregiver	Upper respiratory inflammation	Watch and wait	Symptom improvement
13-2	2	Male	Cerebral palsy	Informal caregiver	Fever	Visiting nurse	Middle ear in- flammation	Continuing antibiotics	Hospitaliza- tion
14	72	Female	Rheumatoid arthritis	No caregiver	Eczema	Visiting nurse	Miliaria	Prescription of ointment	Symptom improvement

^a^CV: cardiovascular.

Remote videoconferencing was conducted in 17 instances involving 14 patients (3 patients were involved in videoconferencing twice). Of the 14 patients, 5 (36%) were children and 5 (36%) were older adults (aged >65 years). Approximately 43% (6/14) of patients were male. According to the International Classification of Diseases 11th categorization, the most common primary disease was that of the nervous system (5/14, 36%), followed by neoplasms (3/14, 21%) and mental, behavioral, or neurodevelopmental disorders (2/14, 14%). About 79% (11/14) of patients lived with family members, 14% (2/14) lived in assisted-living facilities, and 7% (1/14) lived alone.

The reasons for videoconferencing consultation and diagnoses are shown in Table 1. In 77% (13/17) of instances, visiting nurses coordinated the videoconferencing, whereas, in 12% (2/17) of instances, institutional nurses performed that task. In 12% (2/17) of instances, an informal caregiver handled the videoconferencing.

As for subsequent interventions, a new prescription for medicine followed in 35% (6/17) of videoconferences; the physician faxed the prescription to the community pharmacist, and the community pharmacist delivered the new medicine to the patients’ residences. In 18% (3/17) of instances, *watch and wait* until the next planned visit instructions and the guidance on the application of medicine were given. In 12% (2/17) of instances, the physician instructed patients to take the medicine in hand. In contrast, in 12% (2/17) of instances, emergency transfer to hospital was advised; the physician arranged the emergency transfers and passed the patient information to the attending physicians in the hospital who provided emergency treatment.

With regard to outcomes, 82% (14/17) of cases with the use of videoconferencing resulted in improvement of the problematic symptoms before the next planned visit was conducted within the following 2 weeks, and the remaining 18% (3/17) of cases resulted in hospitalization. One hospitalization was because of pneumonia from an upper respiratory infection that occurred independently after the videoconferencing had taken place.

In ID 1-2, although a formal caregiver was available, albeit a part-time worker, the clinic was called, and the physician asked a visiting nurse to visit the patient’s residence as the physician was not able to visit the residence immediately. The visiting nurse subsequently organized and conducted the videoconferencing. In IDs 6 and 8, an informal caregiver was available, and he or she summoned a visiting nurse who organized and conducted the videoconferencing. In ID 7, an informal caregiver was available and organized and conducted the videoconferencing.

### Indicative Videoconferencing Examples

#### ID Number 1-2

The patient was a 97-year-old female with early-stage gastric cancer and chronic heart failure. She lived in a long-term facility with no full-time nurses in attendance but with part-time nurses, with nonmedical informal caregiver helpers providing various care services to the residents. The patient was able to walk using a walker and take a bath with the support of helpers. As she was unable to visit the clinic by herself because of lower-extremity muscle weakness and as her gastric cancer was relatively stable, her medical home care started in February 2015, and the team visited her twice monthly. She wished to spend the rest of her life in the facility. One night in October 2018, the facility's part-time nurse contacted us as the patient had an approximate 2-week history of excess phlegm and breathlessness. The last regular visit by a home care physician was 12 days previously when she had a hoarse voice and was taking regular herbal medicine, as she presumed that she had caught a cold. She was able to eat and did not feel seriously ill; therefore, a home care physician decided to follow up and told her to contact the clinic if her condition worsened. Her body temperature was 37.3 °C, blood pressure was 155/92 mm Hg, pulse rate was 80 bpm, saturation of percutaneous oxygen was 94%, and the state of consciousness was clear. Although a home care physician attempted to visit the facility, the physician was not able to do so immediately. Therefore, the physician ordered a visiting nurse from the clinic to visit the patient’s facility and instead carried out a videoconferencing. The nurse told the physician that there were no obvious lung noises, which suggested that pneumonia was not present, and her body movement, such as standing and sitting, was smooth. The physician carefully observed the patient's complexion and respiratory status through videoconferencing and reconciled the findings with those reported by the nurse. As a result of the videoconferencing examination, the physician ordered the nurse to take a blood test to judge whether there was an acute bacterial respiratory infection and deterioration of chronic heart failure. The nurse returned the blood sample to the clinic and submitted it for testing. The blood test indicated that the white blood cell (WBC) count was 5180 per μL and C-reactive protein (CRP) was 0.12 mg/dL, and the physician diagnosed an acute viral respiratory infection based on the results of the blood test. The physician prescribed a traditional Chinese medicine to reduce her symptoms and then faxed the prescription to a specific pharmacy. The community pharmacist delivered the drug to the patient’s assisted-living facility and started the treatment with the traditional Chinese medicine (syoseiryuutou 9 g 3 times a day for 5 days). Two days later, the physician confirmed an increase in N-terminal probrain natriuretic peptide from the blood test taken by the visiting nurse, and the deterioration of chronic heart failure was diagnosed, and the treatment with a diuretic drug (furosemide 20 mg once per day for 7 days) was started**.** The community pharmacist contacted the patient, delivered the drug to the facility, and administered the first dose. In this case, the nurse being physically present with the patient during the remote examination allowed blood tests to be completed, which allowed the diagnosis of an acute viral respiratory infection and deterioration of the patient’s cardiac problem, thereby allowing early and customized treatment.

#### ID Number 8

The patient was a 16-year-old male with neurofibromatosis type 1. He had a gastrostomy to be fed and underwent noninvasive positive pressure ventilation every night. He spent most of the daytime using a wheelchair and lived in his house with the support of his parents. His twice-monthly home care started in February 2011 to help reduce the burden on his family, who previously had to frequently visit emergency medical departments because of his regular fevers. One morning in September 2018, the patient’s family reported by telephone that the patient had a fever of 37.8 °C with excess phlegm; however, his respiratory condition was not troublesome. As the condition was not serious but needed careful monitoring, the physician told the family to observe with an antipyretic drug and water intake. In addition, the physician ordered a visiting nurse to visit the patient’s residence to check the patient’s condition, as he had acute viral bronchitis with bacterial secondary infection and was treated with an antibiotic 1 month before this consultation. When the visiting nurse visited the patient’s residence that afternoon, his temperature was 37.8 °C, although an antipyretic drug had been administered by his informal caregivers approximately 3 hours previously, and thus, the nurse decided to conduct a videoconferencing. The patient’s condition was similar to that of acute bronchitis that he had experienced 1 month before, which had been resolved following a visit by the team 2 weeks earlier. Therefore, the physician diagnosed acute viral bronchitis with bacterial secondary infection based on the clinical symptoms and the patient’s past history and prescribed a course of antibiotics—sultamicillin tosylate dihydrate 9 g after every meal for 7 days. The prescription was faxed to a specialized pharmacy, and the patient’s mother went to the pharmacy to get the medication and administered it as per instructions. In the meantime, the visiting nurse kept an eye on the patient. The physician performed an emergency home visit 2 days later because the patient’s mother called the clinic as the fever continued. At that visit, as the physician suspected pneumonia, the physician performed a sputum test and a blood test to decide whether his antibiotic drug had to be changed. The results of the laboratory test showed a WBC count of 9410 per μL and CRP of 3.14 mg/dL. The physician judged that his infection was getting past the acute stage as the CRP level was mildly elevated; however, the WBC and other data were within normal limits. The physician decided not to change the antibiotic and finish the 7-day treatment because adolescents with special care needs are prone to viral infections and secondary bacterial infections, and the infections are often severe. The patient’s condition improved 1 week later, and a new bacterial culture of sputum did not reveal any infection. In this case, remote medical examination was useful to diagnose possible acute viral bronchitis with bacterial secondary infection based on the patient’s past history, and action could be taken to respond to the new infection.

#### ID Number 6

The patient was a 35-year-old bedridden male patient who had a postoperative left acute subdural hematoma caused by a traffic accident. He had undergone a number of medical procedures, including a tracheostomy, 2 gastrointestinal stomata, a gastronomy, and insertion of a ventral venous catheter. The two gastrointestinal stomata were used to drain intestinal fluids because he developed nonocclusive mesenteric ischemia and had most of his intestinal tract removed in 2017. For that reason, he underwent a gastrostomy and used a central venous catheter to obtain nutrition. His parents provided his daily care, and home-visiting nurses made two regular visits and provided regular medical attention daily. The clinic also provided weekly medical home care from March 2018. In April 2018, the visiting nurse telephoned the clinic to report that the patient had had fever for a few days, and the physician told the nurse to take blood for testing. From the results—increased WBC (10,210 per μL with a left shift) and CRP (5.65 mg/dL)—the patient was assessed to have an acute bacterial infection. The clinic staff brought sulbactam sodium and ampicillin sodium from the clinic’s stocks to the patient’s residence, and the physician told the nurse to administer it during regular morning and evening visits because there was no allergic information about the patient from the referral hospital. A few minutes after the intravenous injection of the antibiotic, eczema appeared on the patient’s chest, indicating an allergic reaction. Therefore, a remote visual medical examination was conducted to check the patient’s condition. No other abnormal findings were observed, such as respiratory distress or decrease in saturation of percutaneous oxygen, and the eczema disappeared 30 minutes after the intravenous injection. The suspected allergic reaction reminded the patient’s father of the patient’s previous history of suspected allergic reactions, and he reported that the same reaction was observed when the patient was hospitalized and that most of the antibiotics gave the patient redness. As the referral from the hospital stated that the patient had no allergies, the home care physician contacted the hospital; however, there was no information about redness in the hospital records. Thus, they discontinued sulbactam sodium and ampicillin sodium because these drugs were suspected as allergens and instead initiated ceftriaxone sodium hydrate. As such, the remote medical examination was useful for diagnosing possible allergic reactions and helped to change the antibiotics being administered.

#### ID Number 7

A 33-year-old male patient was bedridden because of cerebral palsy and had undergone a gastrostomy. The twice-monthly home care service started in December 2014 because the patient’s family had to regularly take him to a hospital because he was regularly developing a fever. In December 2018, the family called the clinic to report that there was redness in the joints of his left hand, which seemed to be a low-temperature burn. The physician made a remote visual examination using the family’s mobile phone to ascertain whether a home visit was required to deal with the situation. In discussion with the family, the physician confirmed that the red lesion appeared after using a hot-water bottle to keep the body warm. His left hand had been in prolonged contact with the hot-water bottle; however, there was no pain, only redness. Therefore, the physician decided to treat the scald using steroid ointment, which was originally prescribed to be applied to the granulation around the gastrostomy and so was available at the patient’s home, and told the family to contact the clinic if there was any worsening of the patient’s condition. After 2 weeks, the scald healed at the time of the next regular visit. Remote medical treatment was useful to diagnose a low-temperature burn and start treatment immediately without the need for a visiting nurse.

## Discussion

### Principal Findings

Treatments following videoconferencing in the clinic were generally safe and effective. As shown in Table 1, for 77% (13/17) of cases, the problematic symptom causing the need for a videoconferencing improved without emergency transfer or treatment in a medical facility. No intervention by medical professionals was required in 29% (5/17) of instances. The use of videoconferencing was deemed effective for physicians to reduce unnecessary visits, whereas in some cases, more nurse visits were required.

One key element for the successful use of videoconferencing was collaboration between medical staff in the prevailing situation. In Japan, specialized home medical care is overseen by physicians in specialized home care clinics. Other medical staff, such as visiting nurses, community pharmacists, and rehabilitation staff, work in a system whereby the corps of local visiting medical specialists provide more regular home care. A shared notebook stored in the patient’s residence is used to update all clinical information for use by medical professionals involved in the provision of care to coordinate their work and actions. In addition, when medical staff detect any significant adverse signs in the patient’s physical condition while visiting residences, they arrange a teleconference with other medical staff, including physicians, immediately. The videoconferencing enabled visiting nurses to implement prompt blood tests necessary for diagnosis and treatment and subsequently facilitate a physician’s judgment in any necessity for intervention to deal with the patients’ condition. In fact, Funderskov et al [13] showed that the use of video consultation allows community-based nurses to discuss their observations, enabling them to take part in active home care, including palliative care. A comparison of our findings with those in other countries is difficult because of the Japanese health context, where physicians predominantly have governing control in patients’ care. However, in home care and remote examination, physicians have to rely on the judgments and actions of other medical staff to implement timely and appropriate medical interventions.

Videoconferencing allows the analysis of outward appearance of signs and symptoms. Interviews with patients and clinicians in previous studies showed that video consulting was better than telephone consulting, especially in consultations involving psychological assessment where visual cues are important, as illustrated by the cases of IDs 1-2 in this study [14]. With regard to the feasibility and accuracy of diagnosing through videoconferencing, according to a review by Trettel et al [15], most of the articles on teledermatology reported that telemedicine was feasible, reliable, and effective under various conditions. Furthermore, the review noted that in 90.3% of the cases, clinicians rated that the experience of conducting a telehealth session was equivalent to or better than a home visit and that the communication environment (eg, internet reception) was the important factor in telehealth sessions [16]. Japan has a reliable digital communication system: in our cases, all the videoconferencing with the patient’s homes was done using 4G connections, and the communication channel was never interrupted or lost.

However, there are several critical points that need to be considered with respect to incorporating telemedicine in the home care setting. First, the telepresenter, the person who organizes the videoconferencing in the patient’s location, is critical. It is ideal that the main caregivers, including informal caregivers, are able to orchestrate videoconferencing, although this might be a heavy burden for informal caregivers. Clinically, telepresenters are chosen considering their availability, ability, and digital literacy. Future research is expected to clarify whether informal caregivers can play the role of telepresenters appropriately and what kind of support or intervention is required to overcome this problem. Furthermore, in addition to medical literacy, the availability of suitable equipment, technological literacy of patients, their caregivers, attendant nursing staff, and attending physicians, as well as costs, have to be taken into account. A study of telemedicine between nurses and patients at home in Norway highlighted the importance of the levels of training and experience with respect to videoconferencing [17]. In particular, older people are not accustomed to using digital devices, and many do not even own or have access to computers or sophisticated mobile phones. According to Japan’s Ministry of Internal Affairs and Communications, the proportion of internet users in the country is approximately 20% in those aged >80 years, whereas it is >90% in people aged 20-40 years [18]. It might be helpful to instruct all home care caregivers or nurses on how to use communication devices in telemedicine. A previous study recommended supporting digital literacy and confidence of older people to strengthen their intention to use technology [19]. Considering the increase in the number of older patients who live alone in Japan, developing digital skills among older adults is also beneficial to create more space in the limited caregiving facilities. Therefore, policies may have to be put in place to accommodate the provision of medical home care to older patients who live alone.

Second, the cost of communication devices should be considered. Some patients do not have any equipment enabling videoconferencing, which poses significant constraints on developing a videoconferencing telemedicine system. This endangers one of the primary aims of videoconferencing implementation—equity. In fact, the Ministry of Internal Affairs and Communications reported that the internet use rate in Japan was >54.2% in households, with an annual income of <¥2 million (<US $19,000), whereas it was >85% in households with >¥4 million (>US $38,000) [20]. The cost of internet use depends on the type of service used. For example, in cases of our study, because the smartphones of patients, family caregivers, or clinics were used in videoconferencing, no additional cost other than communication costs paid by patients was needed. Commonly in Japan, medical institutions use remote examination platforms or phone calls when conducting remote examinations [21]. However, it is uncommon that the Japanese national insurance system covers the cost of provision of adequate devices, training in their use, the videoconferencing connection, and the administration of setting up videoconferencing. It is important to encourage patients or caregivers to, wherever and whenever possible, be able to use modern communication equipment, enabling them to use videoconferencing or telemedicine in times of emergency or whenever a physician is unable to make a physical visit. Another option is to change the system so that nurses and paramedics can conduct medical interventions without referring to physicians.

Third, telemedicine may miss some important clinical information that could be obtained in a face-to-face consultation. In addition, orchestrating sample collection, transport for testing, and analyses also takes longer than it would in a hospital or clinic environment, and the inability to use specialized equipment to aid diagnoses, which would often be available in medical facilities, makes home care less than ideal. Of note, we should be aware that the use of telemedicine in emergency cases in a home care setting is still in its infancy, and a careful and gradual evolution is imperative. It is essential for physicians and other health care professionals to perform a follow-up, in-person evaluation following any telemedicine event, as is currently done. It is also important to err on the side of caution when carrying out emergency telemedical consultations by calling for emergency transfers in cases possibly requiring rapid interventions or access to specialized physicians, facilities, or equipment.

### Limitations

The sample size of this study was small and diverse, and the study findings should be interpreted with caution. We could not compare the difference in the feasibility of conducting videoconferencing, depending on the living environment; however, formal caregivers may facilitate videoconferencing better because of their knowledge and experience of using videoconferencing. Moreover, considering the increase in older patients who live alone in Japan, the costs involved, and the scarcity of suitable accommodation, it is difficult for them to move into caregiving facilities. Therefore, new strategies and policies should be developed to ensure the provision of a suitable level of care as Japan moves toward increased provision of home care for patients.

Owing to the notification from the MHLW concerning remote examination, it is expected for videoconferencing in home care to increase in Japan in the foreseeable future. Troubles may manifest when considerably more examinations are conducted at the same time. In particular, as we discussed, the age of patients and caregivers can affect the use of videoconferencing, as observed in this study. Future research should be conducted with larger sample sizes to examine the reliability of our findings through stratified analyses.

### Conclusions

Consultations using videoconferencing in clinics are generally safe and effective**.** Telemedicine is a valuable substitute for face-to-face investigations where necessary or desirable. This is a particularly valuable option in emergency situations, especially when home care health personnel cannot access the patients under their care. Japan has recently introduced the concept of primary care and has introduced training and qualifications in primary care. This will have a major impact on home care options and practice, and the importance of and potential for videoconferencing will be further enhanced accordingly. Furthermore, the findings of our study have special implications in the COVID-19 era. Considering the vast and long-lasting impacts of the current global COVID-19 pandemic, where physical visits are restricted, the findings in this study can help health care workers, patients, and their families involved in home care to appraise the characteristics of this relatively new approach.

## Abbreviations

CRP: C-reactive protein
MHLW: Ministry of Health, Labor and Welfare
WBC: white blood cell
